# Carrying out Physical Activity as Part of the Active Forests Programme in England: What Encourages, Supports and Sustains Activity?—A Qualitative Study

**DOI:** 10.3390/ijerph16245118

**Published:** 2019-12-14

**Authors:** Liz O’Brien

**Affiliations:** Forest Research, Social and Economic Research Group, Farnham GU10 4LH, UK; liz.obrien@forestresearch.gov.uk; Tel.: +44-300-067-5700

**Keywords:** physical activity, forest, qualitative research, social practices, mental wellbeing

## Abstract

The Active Forests programme was developed through a partnership between Forestry England and Sport England. A three-year pilot programme focused on five forest sites ran from 2014. It was mainstreamed in April 2017 and is now running on eighteen forest sites in England in 2019. The aim of the programme is to encourage a physical activity habit, and participants can get involved in a wide range of activities from Nordic walking to mountain biking, Pilates, running, and Bootcamp in different scenic forests. The aim of the research was to identify the motivations, benefits and the overall experience participants had from their forest physical activity. As part of the programme, qualitative data was gathered through in-situ participant observation, and interviews or focus groups. One hundred and twenty people were involved in the research. The findings identify some of the key elements of the programme that encourage, support and in some instances help to sustain or change physical activity. These include participants gaining a wide range of wellbeing benefits; appropriate targeted activities; opportunities for progression; social connections; providing challenge; and a supportive atmosphere. There is evidence of participants sustaining and changing physical activity practices, however attribution of this to the Active Forests programme is not straightforward. The programme is also explored through the lens of social practice theory and its concepts of materials, competence, and meanings. The research highlights how a targeted physical activity programme can reach and involve a broad range of people from the already physically active to those who are less active.

## 1. Introduction

The British Heart Foundation states that physical inactivity is a global health crisis [1]. Approximately 39% of United Kingdom (UK) adults are not meeting the recommendations of physical activity which is 150 min of moderate to vigorous intensity activity per week and two or more days of strength activities. Women are more inactive than men with 36% more likely to be classified as inactive than men [1]. The direct financial cost of physical inactivity to the UK is estimated to be £1.2 billion each year [1]. Physical activity can help to reduce cardiovascular disease, type II diabetes, colon, and breast cancer, depression and dementia [2]. Heath et al. [3] in a review of physical activity interventions found that programmes that promote physical activity can increase effectiveness if health agencies form partnerships and coordinate with several organisations; if environmental spaces can be created or enhanced to be conducive to access for physical activity; and if programmes increase social support for physical activity. The National Institute for Health and Care Excellence provide national guidelines and advice to improve health and social care. Its guidelines on physical activity, particularly walking and cycling, suggests that interventions to improve this type of activity should comprise an integrated package of measures and incorporate actions in specific settings; include communication strategies to publicise opportunities; and motivate people to use them; and it suggests that these should be evaluated [4]. There are strong links between physical and mental health, a number of studies for example have found that physical activity is negatively associated with anxiety and depression disorders [5,6,7]. Previous research suggests men are motivated to be more physically active by competition and challenge [8], while women are more motivated by improving appearance [9,10], and loss or reduction in weight [10]. Certain sports and the environments where they take place, such as a gym, can feel intimidating to women [11]. Motivations can also differ by age. Research on motivations across the lifespan [12] found that friends, fun and mental toughness were motivators for young age groups, while being fit, toned and reducing stress became important motivators with increased age. A study in Australia found that men in middle age were motivated by disease prevention and weight management [13]. According to WHO Europe [14] every year in the WHO European region an estimated one million deaths are caused by physical inactivity. Thompson Coon et al. [15] found that exercising in natural environments was associated with a decrease in tension, anger, confusion and depression and an increase in energy when compared to indoor exercise. Public Health England ([16], p.4) outlines ‘if being active was a pill, we would be rushing to prescribe it’. It also identifies that men are more active than women in England in virtually every age group [16]. The health benefits of nature have been identified in a wide range of studies in recent years [17,18,19,20,21,22]. In a synthesis of thirty-one studies on the wellbeing benefits gained from woodlands and forests; women, and those aged 35 years and over identified a wider range of benefits than younger age groups [23].

### 1.1. The Active Forests Programme

**Figure 1:**
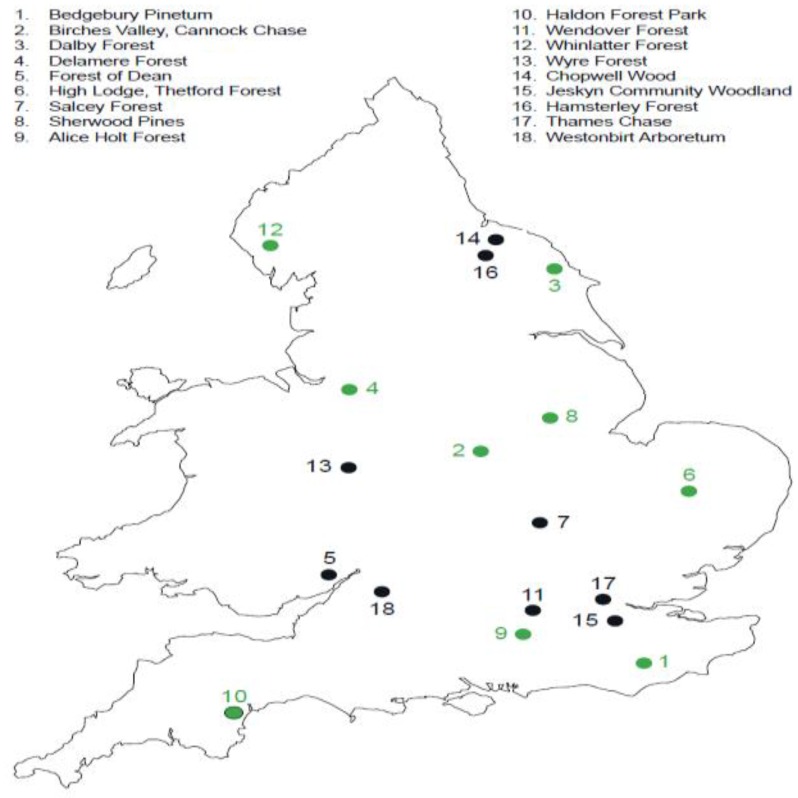
The Active Forests sites in England. Green dots show the forests included this study.

**Figure 2:**
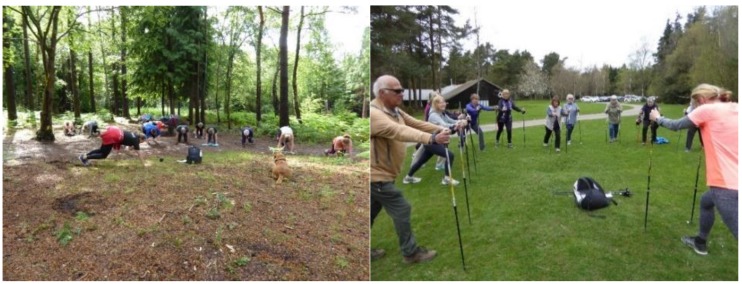
Bootcamp participants at Alice Holt Forest and Nordic walkers at Haldon Forest Park.

The Active Forests programme started in 2014 and was developed in a partnership between Forestry England and Sport England. Forestry England is the country’s largest land manager and largest outdoor recreation provider, shaping landscapes and enhancing forests for people to enjoy, wildlife to flourish and businesses to grow. Sport England is a non-departmental government body under the Department for Digital, Culture, Media and Sport, its focus is on developing an active nation where everybody feels able to participate in sport and be active. In recent years, Sport England has included a focus on outdoor recreation and has started to work with a range of environment sector bodies to encourage physical activity in nature. A review of outdoor recreation commissioned by Sport England identified different types of people who want to spend time outdoors and this included those who want to explore the natural world, challenge themselves outdoors, and those interested in fresh air and freedom [24]. The Active Forests programme was piloted from 2014–2017 on five sites in England [25]. Due to the success of the pilot the programme was mainstreamed in 2017 and now runs on 18 forest sites in England (Figure 1, Figure 2, and Appendix A). Each site has an Active Forests coordinator whose role is to work with local sports clubs, local communities, and develop activities that are appropriate to that forest site. The physical activities and events are generally delivered by a range of third-party providers for example a Nordic walking instructor, a Pilates instructor or local running clubs. Figure 1 shows the location of the sites in England. Table 1 outlines the key elements of the Active Forests Programme which shows a multi-pronged approach with improvements to forest infrastructure particularly forest trails, the provision of equipment, organised activities led by instructors or volunteers, and the provision of information and communication to highlight what is on offer. Table 1 also shows some of the variety of physical activity opportunities that people can take part in and these can be regular activities led by trained instructors or volunteers; they can be self-led activity for example, people taking advantage of a new running trail; or there can be events that happen once or twice a year that people can sign up to in order to try something different or step up to a challenge. This research sought to address the following questions:

- What are the motivations and benefits or disbenefits of undertaking physical activities in forest environments through the Active Forests programme?
- Is there evidence of sustained or changed physical activity behaviours being realised from participating in the Active Forests programme and what supports this?

### 1.2. Theoretical Perspective

This research draws on social practices theory which focuses on behaviour as an ‘observable expression of social phenomenon’ [20]. In this conception individual behaviour is the tip of the iceberg and beneath it lies three elements: materials, competence, and meanings [26,27,28]. In the Active Forests programme the materials include the forest trail infrastructure created or improved through the programme, the equipment that enables specific activities to take place such as bicycle hire, table tennis tables and the bat and balls that go with them, equipment for volleyball, football and rounders for families and friends to play. Competence includes the knowledge and skills of people to undertake physical activity, to get involved in specific activities such as mountain biking, cycling, playing volleyball, as well as the competency and knowledge to be able to know where to find out about opportunities for physical activity in a forest environment. Finally, meanings refer to cultural conventions and socially shared meanings. For example, the success of parkrun and the rise of approaches such as ‘couch to 5K’ (an approach that helps people to plan to become active and run 5 kilometres over a specific period of time) are changing the cultural convention of running as a practice for athletes and very fit individuals to an activity that can be undertaken by those of a variety of ages and abilities and is therefore more inclusive as a physical activity practice. In these newer approaches all are welcome to become involved and encouraged even if they walk all or part of the route while others run. Previous research suggests that access to forest environments for a variety of activities is linked to shared meanings of escape and freedom from everyday busy lives and concerns, fresh air, and contact with nature providing an opportunity to reflect and restore wellbeing [29,30,31].

## 2. Materials and Methods

**Figure 3:**
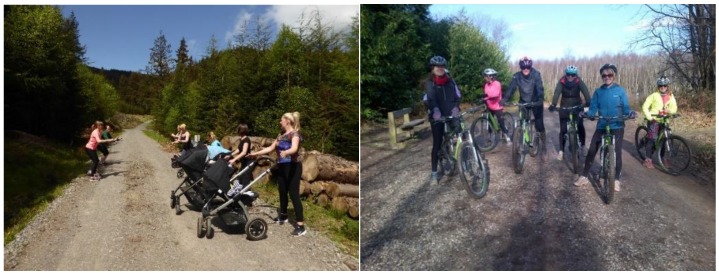
Buggy Fit participants at Whinlatter Forest and Real Spin participants at Begebury Forest.

**Figure 4:**
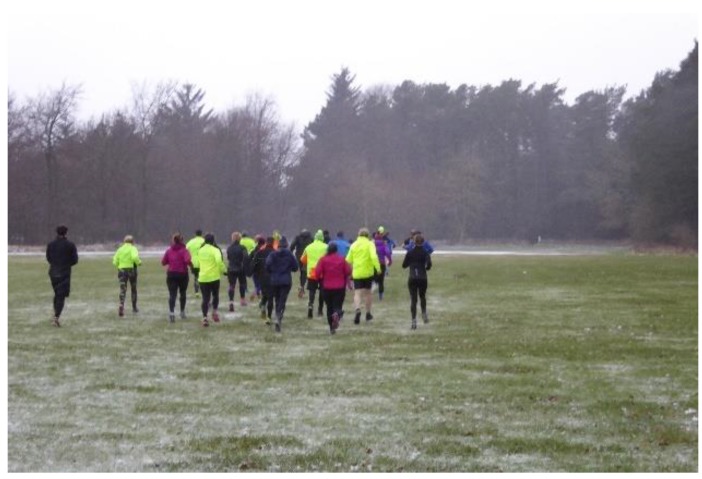
Go Tri duathlon participants at Dalby Forest.

This research is part of a wider evaluation of the Active Forests programme which includes a mixed methods approach [25,32]. This paper draws exclusively on the qualitative evaluative material. In-situ participant observation was undertaken by carrying out the physical activity (where this was possible) with participants to observe how the activity was organised and how it worked, how it was led, the types of people getting involved, and how people interacted together before and during the activity (Figure 3 and Figure 4). After the activity, participants were involved in an interview or focus group (Appendix B). The method allowed flexibility, for example at the Go Tri (an approach to introduce beginners to triathlons and duathlons) event at Dalby Forest, only short interviews were undertaken as the event was held in winter, when it was snowing, cold and windy. There was limited shelter for participants when they ended the race on top of a hill in a large field. Therefore, short interviews were only possible before participants needed to get warmed up. At the parkrun event at Sherwood Pines Forest, participants who were happy to be involved in the research were invited into the forest classroom after the event. However, participants finished at various times and would enter the classroom when they finished. A short focus group was started and was then followed by individual interviews. The table tennis activity at Thetford Forest involved the researcher observing the activity over a four-hour period but not getting involved in the activity itself; rather interviewing the parents, relatives and carers of the mainly children who got involved in playing table tennis. In total, 120 people were involved in an interview or focus group (Table 2).

The sample chosen was a purposive one to include data gathering at: irregular or one off events such as the canicross and Go Tri events; as well as self-led activities such as table tennis; and regular led activities—led by a trained instructor—such as Nordic walking or Pilates; or led by volunteers such as parkrun [33,34]. Data was captured from five forest sites in the pilot project and from six forest sites in the mainstream programme, so across nine different sites in total to cover different areas of the country with a variety of populations surrounding the sites. Groups to be studied were identified through discussions between the researcher and the Active Forests programme board. Active Forests coordinators were able to contact the groups to inform them about the research and seek permission for the researcher to attend one of their activity sessions and to participate in the activity and follow up with data gathering. The focus groups and interviews explored key areas including: when participants got involved in sport and physical activity, how much they get involved in physical activity in a forest environment and why, their experiences of carrying out their physical activity in a forests, any sustaining or changes in physical activity practices, the role of social connections, and engagement as part of their forest experience. The interviews and focus groups lasted from between ten to sixty minutes. Short ten-minute interviews took place at the Dalby Forest canicross and Go Tri events due to poor weather and the lack of a nearby place for participants to take shelter while being interviewed.

Informed written or verbal consent was gained from all focus group or interview participants. All data was anonymised and no names of participants was used. An ethical code was used as a guide to developing the research and gaining informed consent [35].

### 2.1. Data Analysis

All of the interviews and focus groups were transcribed and participant observation field notes were also captured. The data was imported into NVivo12 (qualitative data software package, QSR International, Doncaster, Australia) which was used to categorise the data and then to explore the data in detail through coding segments of text and observational notes. Coding is an important part of the analytical process and the codes were combined into overarching themes as part of the interpretative analytical approach. An inductive approach was taken with the themes that were identified strongly linked to the data [36] which focused on exploring the experiences and meanings of the participants involved in the programme. The themes identified capture important aspects in relation to understanding what motivates people to be active in a forest environment, what can enable that to happen and if there are any benefits that people gain from their activity, and if any sustained or changes in behavioural practices occur.

### 2.2. Limitations of the Research

There were some limitations to the research, for example, only short interviews were undertaken at the Dalby Go Tri and canicross events partly due to the weather conditions, but also because there was no shelter nearby to hold a focus group or interviews. A very short focus group and then individual interviews were undertaken at parkrun because participants finished their runs at different times, making it difficult to hold a longer focus group. It would have been useful to identify more specifically how physical activity fitted into people’s everyday lives and the variety of places in which people undertake their physical activity beyond the forest environment. However, this did not occur in detail due to the short interviews and focus groups with some of the participants. More interviews and focus groups were undertaken with women because it is an important focus within the Active Forests programme to reach out to women who are often less active than men. Furthermore, some activities such as the Nordic walking seemed to attract more women than men. This means that there is less evidence in the results on men and their physical activity in forests.

## 3. Results

The results are presented under the key thematic headings of motivations, enablers, benefits and practices; links to social practices theory are identified in the discussion section.

### 3.1. Motivations to Be Physically Active in Forests

The evidence highlights that motivations for people to be physically active in a forest environment and the benefits they gained were often very closely linked. For example, if people were motivated to get fitter then one of the benefits was often participants stating that they felt fitter.

The motivations to participate in the Active Forests activities included health problems and health reasons. For example, health problems included those related to a specific health issue faced by an individual such as asthma, knee or back trouble, depression, diabetes or heart problems and recovery post-surgery.

I have done a few runs and duathlons and stuff and this is my way of getting back into it, because I am in the middle of chemo for bowel cancer at the moment. So this is a good way of getting out into the fresh air. If I can do it anyone can.

(Male, Go Tri, Dalby Forest)

While health reasons were linked to preventing ill health and a desire to improve health by losing weight, getting fitter, improving mobility, aging well, or a desire to avoid hereditary health issues within the family, such as heart disease.

Life transitions such as having children, after children have grown up, retirement, redundancy or changes in work patterns to part time work or shorter working hours or getting older could also act as a motivational catalyst to take up a new activity, start to be active, or increase existing levels of activity.

Interviewer: *you said it was your first time here, what encouraged you to come out and do Buggy Fit.*

Participant: *To get my body back and to meet new people.*

(Female, Buggy Fit, Whinlatter Forest)

So, when my children grew up my youngest one at that point was eighteen, so I decided to do stuff for myself. I have always taken then to rugby, ballet dancing, fitness but never thought about myself.

(Female, Go Tri, Dalby Forest)

For women in their 50s at a Bootcamp session there was acknowledgment of the need to ensure that they started to consider strength exercises in their overall activity regime to support healthy bones and prevent osteoporosis.

*I think the mental health side is huge, but also I am really conscious as a woman of a certain age we aregetting to a point where strength work is really important.*

(Female, Bootcamp, Alice Holt Forest)

While women at a mountain biking class called ‘Real Spin’ were motivated to join a women’s class to reduce the pressure they thought they might face from a mixed gender class:

Female 1: *Men are too competitive and change the dynamic and the conversation.*

Female 2: *And they bump you off the trails, they are behind you with their breaks on.*

(Females, Real spin, Bedgebury Forest)

Other motivations included signing up to a specific event such as a duathlon or 5 or 10 km run, taking up a challenge, or joining an activity class to undertake exercises people thought they would generally not undertake on their own.

*‘Running you can just do from your front door, but I’d never do this sort of exercise* [e.g., star jumps, press ups etc.], *so you need a bit of a class’.*

(Female, Bootcamp, Alice Holt Forest)

Spending social time with others such as family and friends was also an important motivation. For example, participants talked about being able to spend time carrying out activities with children; in particular as a way to get them off their gadgets and out into the fresh air. A few people talked about being inspired by others to take up an activity. A woman in the Nordic fitness walking class persuaded a friend to join her:

Female 1: *It’s taken me two years to persuade her actually*

Interviewer—*what finally tipped you into coming*

Female 2: *I couldn’t put her off any longer, but now I wonder why I didn’t do this sooner. So I’ve enjoyed it a lot more than I anticipated.*

(Females, Nordic fitness walking, Haldon Forest)

Spending time with their dogs was a motivation for those getting involved in a canicross event (running with dogs) at Dalby Forest. For a small number there was a desire to act as a role model for children by being active and people also outlined how they were inspired by others to become active.

Female 1: *My son started mountain biking at school ‘cause he wanted to follow what I was doing*

Female 2: *It’s good for your children to see you going out and doing things, you get out of your comfort zone.*

(Females, Real Spin, Bedgebury Forest)

*I’m trying to get back into being active after a gap, to encourage the children to have active lifestyles.*

(Male, Parkrun, Sherwood Pines Forest)

### 3.2. Enablers to Being Physically Active in Forests

A key objective of the Active Forests programme is to encourage and enable physical activity on the 18 forest sites taking part. Forestry England has improved forest site infrastructure and information and communication to provide a suitable environment for physical activity and to inform people about what is on offer (Appendix A). Participants in the interviews and focus groups identified a range of enablers. For example, those getting involved in regular led activities highlighted the important role of the instructors leading the activities. The instructors were trained in whatever activity they were leading such as Nordic walking, Pilates, etc. They worked to build relationships with those attending their sessions and tailored activities to people’s abilities. Women in the Buggy Fit group, where they bring their babies in pushchairs, outlined the importance of their instructor being trained in post-natal exercise programming. The instructor could support the women who had recently given birth in undertaking appropriate physical activity.

Cause J is trained in post-natal fitness it is much easier going to her—someone who understands a lot of the problems you get during and after pregnancy. It makes it a lot easier than just going to an exercise class and they don’t know what to do or exacerbate the problem.

(Female, Buggy Fit, Whinlatter Forest)

Trained instructors could also help participants to set fitness goals, this was the case with the Nordic fitness walking group at Haldon Forest whose participants were in their 50–70s. Instructors were also able to adapt exercises for those who had an injury, and were seen as key in enthusing participants and changing the activities participants were doing, as the following outlines:

You never find yourself looking at your watch and thinking when’s it end. I love it because it’s different all the time.

(Female, Pilates, Delamere Forest)

Providing participants with challenge and ensuring that activities are varied were also enablers for participants to take up activity along with meeting others and joining group activity at a specific time every week.

Oh yes you have to keep it up, we go twice a week.

(Female, Pilates, Delamere Forest)

Activities that were perceived to be inclusive due to their ethos and atmosphere also enabled people to take up activity they may not have done previously. Parkrun which is led by trained volunteers aims to be inclusive and encourage everyone to walk or run the 5 km courses. The people who attend cover a range of ages, and abilities. Being able to hire equipment or use free equipment also enabled some to participate easily. For example, bats and balls were provided with the table tennis tables making it easy for families to stop and have a short game before moving on to other activities.

*This is the great thing about this place you can spend the whole day and if they* [the children] *get bored you can move on to the next thing.*

(Male, Table tennis, Thetford Forest)

One woman in the Real Spin class talked about being able to use the cycle hire facility on site and how convenient that was.

I also love the fact that you can just come, you don’t have to worry about bringing your bike. That is a huge advantage because I used to bring my own bike but there is lifting it in and lifting it out of the car.

(Female, Real Spin, Bedgebury Forest)

The quantitative Active Forests programme data reveals that the majority of people hear about the opportunities for activity through word of mouth [19,26]. A woman who got involved in the Buggy Fit class found out through ‘netmums’ which is a website for parents in the United Kingdom. Finally, an enabler mentioned by women in the Bootcamp class was that that the forest environment was perceived to be a less judgemental space than exercising in the gym or running along roads. How you looked or were dressed and how fit you are or not seemed to be less of a pressure in a forest environment for some of the women in particular.

I think the reason so many women like running in the forest is there are no vans, no cars, no one can see you, no blokes, there is no car going beep beep.

(Female, Bootcamp, Alice Holt Forest)

Other women who were older in the Nordic walking classes also talked about the gym as an intimidating space, outlining that it was often full of younger people in tight sportswear.

### 3.3. Benefits of Being Physically Active in Forests

There were many benefits identified by participants from being physically active in a forest environment through the Active Forests programme. Mental wellbeing benefits came out strongly in the evidence with participants talking about gaining stress relief from being active, the forest being a calming place and the enjoyment and fun they associated with being active in a forest (see also Table 3).

Female 1: *I think that is the nub, mental health even more than physical activity*

Female 2: *I run out my crazy—that is what I tell people.*

(Females, Bootcamp, Alice Holt Forest)

‘I’ve never really had a problem with motivation it’s just I’ve had a really bad history of depression when I was younger which still comes back from time to time and the only sure fire success for me is running, it always lifts my mood’.

(Female, Parkrun, Sherwood Pines Forest)

If I get out there is an immediate impact so it can help with day to day living and mental health.

(Female, Real Spin, Bedgebury Forest)

I find running relaxing if I’ve had a hard day at work, I have a hard run which is relaxing.

(Male, Orienteering, Cannock Chase Forest)

Social interaction could also be important, and this could be with new people when someone joined a group or an event or it could be with friends and family. One woman found cycling with others gave her more confidence and support.

*That’s why I started coming cause I came on my own and then I fell off and it’s a bit miserable falling off on your own and that is why I joined the group.*

(Female, Real Spin, Bedgebury Forest)

Participants did not need to be on their own or live alone to want to join a group and enjoy the company of other people.

D and I are retired so it’s nice to come out and meet other people even for just an hour or so. Even in the winter when you wouldn’t really go out, it gets you out of the house.

(Male, Pilates, Delamere, Forest)

Exercising with others in the family was a benefit for a number of participants. One woman talked about carrying out parkrun with her niece, parents talked about running with their children at parkrun. Grandparents outlined how they could play table tennis with their grandchildren. A mother in law and her daughter in law explained how they carried out physical activities together rather than going for a coffee. When interviewed they had just participated in the Go Tri duathlon at Dalby Forest. The activities provided the opportunity for people to spend time with others. Meeting new people by joining an exercise group could lead to new friendships. A couple of women undertaking Pilates at Delamere Forest highlighted that this is how they met. Getting to know people in a group also helped motivate people to continue in that activity because if they missed a session, others would notice they were absent and ask them about this the next time they met.

Physical benefits were also important for example, those who had a health issue could still find ways to be active or prevent problems such as back pain.

I was recommended to do this by a physio as I have a really bad right knee and I was running but she said try cycling as its low impact. I didn’t imagine it was but it’s been great. Yes my knees are so much better definitely.

(Female, Real Spin, Bedgebury Forest)

Its particularly about strength, core strength and flexibility particularly for people with back problems.

(Male, Pilates, Delamere Forest)

I’m a lorry driver by trade, so all week I’m sat on my backside running up the country, so I’m trying to cram everything in to 2 days on a weekend. I’ve just completed it in just over an hour—so that will do.

(Male, Go Tri, Dalby Forest)

Carrying out specific types of physical activity could also provide young people with the opportunity for skills development, as the following quote suggests:

It teaches self-reliance, you might be on your own in the woods with a map but kids have to sort it out and these are life skills. The skills translate to other things.

(Male, Orienteering, Cannock Chase Forest)

The forest environment played an important role in people’s physical activity. This related to sensory benefits such as seeing wildlife, and enjoying the scenic nature of the forest sites.

The forest is very varied, so you always have an interesting view and on a hot day you have the dappled shade as well which is lovely.

(Female, Nordic fitness walking, Haldon Forest)

*It’s on my doorstep, I’ve been coming up here all my life. There’s been a huge change* [in the forest] *but for the good. I think it brings people in, the facilities up here now are brilliant and it’s great, it gets people outside and that is not a bad thing.*

(Male, Go Tri, Dalby Forest)

Participating in an organised session could also enable people to visit parts of the forest sites they had not been to previously.

I thought I knew the forest but. there are so many paths we go on, it’s absolutely wonderful.

(Female, Nordic walking, Delamere Forest)

Participants talked about the atmosphere of the forest sites with opportunities to carry out their activity in scenic and attractive settings, to see changes in the seasons and spot wildlife. Many participants involved in regular led activities or events talked as well about the atmosphere of the activities as welcoming and supportive, with people being friendly and approachable.

Here it’s a nice atmosphere and the scenery is really great. It takes us 25 min to get here but it’s a nice place to come.

(Male, Parkrun, Sherwood Pines Forest)

There are changes in the seasons, you are more observant of what is around you. You look and listen for things.

(Female, Nordic walking, Delamere Forest)

### 3.4. Disbenefits

Although there were many benefits identified by participants of getting involved in the Active Forests programme, a smaller number did identify negative issues. The most prevalent of these were the costs of car parking, particularly when participants were also paying to join a regular session led by a trained instructor such as Pilates or Bootcamp. Even paying for parking on its own some felt was expensive particularly if participants visited the forest sites regularly such as every week. Forestry England has what is calls a discovery pass for its major forest sites which visitors can buy for an annual fee. It then gives visitors free parking, information and news about events and activities they can join. However, while quite a few participants were aware of this and had these passes, a couple mentioned that it could not be used across different forest sites; it was only linked to one site. Another issue that participants sometimes found problematic was how busy some of the forest sites would get at weekends, in the school holidays and at bank holidays, when getting a parking space might be difficult or getting a drink or something to eat from the onsite café meant potentially queuing for quite a long period of time. A small number stated that the forest sites were too far away from where they lived to use regularly, a couple of people felt that there should be public transport available to reach the sites. One woman at Thetford Forest whose children were playing table tennis stated that sometimes her children could not get onto the tables as they were so busy. While another woman welcomed led and organised activities because, as she outlined, she had a:

‘Fear of people, there are some very strange people in the forest sometimes’.

(Female, Nordic walking, Delamere Forest)

### 3.5. Sustaining or Changing Physical Activity Practices

The Active Forests programme aimed to encourage those who were meeting the physical activity target of 150 min a week of moderate to vigorous activity to sustain that activity while trying to enable those who were not meeting the target to do more. The quantitative data [19] showed a statistically significant increase in those who were less active (less than 2.5 h per week) stating they were more active at their three month follow up. Those who were doing more activity were asked whether they were doing this activity in the forest, in the forest and elsewhere, or elsewhere; and approximately 46% stated they were doing more physical activity both in the forest and elsewhere. The qualitative data reveals that some people liked to move to indoor physical activity in the winter. In the quantitative data it was difficult to identify if increases in physical activity were directly attributable to the Active Forests programme. The qualitative evidence also highlighted this difficulty. For some people it was obvious that the Active Forests programme had been the key instigator for changes in physical activity behaviour:

*My first* run *was 12 weeks ago. It’s made a massive, massive difference. I’ve lost one stone and nine pounds and I’ve come off anti-depressents. It just keeps me going. I come* [to Parkrun at Sherwood Pines] *with my niece, I never thought my niece would want to come with me. So now we come every week and have aunty and niece time together’.*

(Female, Parkrun, Sherwood Pines Forest)

For others physical activity waxes and wanes over their lifetime, and life transitions can act as a catalyst to become more physically active. For example, some of the women talked about taking up physical activity once their children had left home or grown up and they had more time for themselves. For younger women, getting active again after giving birth was important to get back into shape and get out of the house with their baby as the following quotes suggest:

Interviewer: *What motivated you to join Buggy Fit?*

Female 1: *I can’t spend all day in the house with her* [the baby] *it would drive me mad*

Female 2: *I was really depressed when I first had the baby as I thought I was never going to walk up hill again.*

(Buggy Fit, Whinlatter Forest)

Reaching different milestone ages could also act as a catalyst for more activity:

I didn’t start doing any fitness until I was thirty. I was three stone heavier than I am now, drank a lot, eat a lot. Since then I have been in and out of running’.

(Male, Parkrun, Sherwood Pines Forest)

I’ve got more into fitness in recent years, as you get older it’s about looking after yourself.

(Male, Go Tri, Dalby Forest)

Retiring or reducing working hours could also mean that people were looking for something else to do or to extend what they did already. Joining an exercise group could also help people to get to know the forest sites better and use footpaths and areas of the forest they had not done previously, which they then could go on to do with friends and family.

Getting involved in an activity could inspire people to take up challenges. A woman who participated in the Real Spin mountain biking sessions had been inspired to undertake a cycling holiday one year and then rode from London to Paris the next.

‘Yes without a doubt the Real Spin has inspired me’.

(Female, Real Spin, Bedgebury Forest)

## 4. Discussion

The Active Forests programme in mid-2019 has been running for nearly five years, it is a major intervention for Forestry England across eighteen of its forest sites. The key results provide evidence of the close link between motivations to be physically active and the benefits that people gain. The benefits can also provide the motivation to return and to continue being active [15]. The research illustrates that physical activity in attractive Active Forests environments can lead to a wide range of wellbeing benefits that go beyond the physical. Both men and women talked about benefits associated with mental wellbeing, and social interaction, as well as enjoying being outdoors in sensory rich environments. These benefits are similar to those found in previous studies [17,18,19,20,21,22]. This research found differences in motivations for being physically active with women outlining life transitions, strengthening bones for middle aged women, reducing feelings of pressure by being involved in women focused groups, and the social opportunities to be with friends, meet new people or spend time with children. Fewer men were involved in this study and they were motivated to be active for health reasons, getting back into activity after a break from exercise, or injury, personal challenge and competition, developing skills and problem solving. However, both men and women talked about being motivated to be active for health reasons. This has some similarities with previous research which suggest women’s motivations focus more on appearance and physical condition [8,10,37]. However, this research importantly found that the forest environment was seen by some of the women as a space where they are not judged on appearance and where they can feel more comfortable exercising and less self-conscious. Much of the research on motivations to be physically active is quantitative and qualitative approaches such as this can add further insights into how and why people engage in physical activity and the important role the forest environment can play in supporting physical activity.

The partnership with Sport England has provided an opportunity for Forestry England to contribute to the governments ambition of encouraging a greater number of people to become more physically active and to sustain existing recommended activity levels [14,16]. The pilot programme which ran for three years on five sites enabled Forestry England to trial and test different approaches and look at what worked and what did not, before it mainstreamed the programme. Due to the size of the programme and each site having a dedicated Active Forests coordinator, whose role it is to seek out new opportunities for people to be active, the programme has created a range of options aimed at different age groups, with varying abilities and levels of fitness. Therefore, there is the opportunity for people to get involved in a way that suits them and fits in with their everyday lives which can be extremely busy; or it has enabled them to try something completely different. The disbenefits identified were anthropogenic with no mention of any issues such as those concerning wildlife, or ticks. However, some studies on ticks (which can cause Lyme disease) for example show that while these issues are of concern, they are often placed in a wider context by those engaging with forests [38,39].

### 4.1. Social Practices and the Active Forests Programme

From a practices perspective, the motivations identified by participants were strongly linked to the social practices concept of ‘socially shared meanings’ such as the concept of aging well through being physically active, the importance of being mobile in later life and maintaining health. This was emphasised as important by both men and women. There were also shared meanings connected to life transitions leading to more time for participants to take care of themselves and consider their own needs. Increasingly people are looking for challenges that they can undertake. Parkrun recognises this need for affirmation or reward from meeting a challenge, and it provides T-shirts for those who have reached specific milestones such as running 50 or 100 parkruns. As health promotion and ill-health prevention become increasingly important expectations about the need to keep fit has risen across the health sector and into wider cultural discourses such as being active to reduce the chance of getting dementia, heart disease, type 2 diabetes, and certain types of cancers [2,14]. Finally, meanings were strongly linked to the benefits people stated they gained from engaging in the various physical activities such as mental health, social engagement, the atmosphere of the forest sites both in terms of the scenery but also the friendliness of instructors and other participants. The forest sites were also seen, particularly by women, as places where they are not judged in terms of their physical activity ability and their appearance in a way they felt they would be in a gym environment [40,41].

The social practices concept of ‘competence’ was also relevant to motivations and to participants wanting to know more about how best to exercise and how to improve or recover after a health problem. Participants also outlined that they learnt about physiology from some of the instructors leading the activities. Competence could also be an enabler of activity with some participants knowing where to look to find out about new opportunities at the forest sites or finding out from families and friends about activities they could get involved in. Active Forests coordinators worked to develop activities that might appeal to different groups and instructors of led activities and events worked to match the activity to people’s abilities. Targeted promotion of opportunities by Forestry England was used to reach out to those who might not be so familiar with accessing and enjoying forest sites.

Approaches that enabled the Active Forests activities to take place were strongly linked to the social practices concept of ‘materials’. For example, the improvement of the forest infrastructure was a critical part of the Active Forests programme with improvements in trails, development of new trails, and linking with locally trained instructors to provide a wide variety of regular weekly activity sessions; these all help to influence where physical activity can take place. The equipment provided also allowed for new activities to take place in the forests such as table tennis, volleyball etc. These were activities not previously provided at any of these forest sites.

### 4.2. Moving beyond a Focus on Individual Behaviours

**Figure 5:**
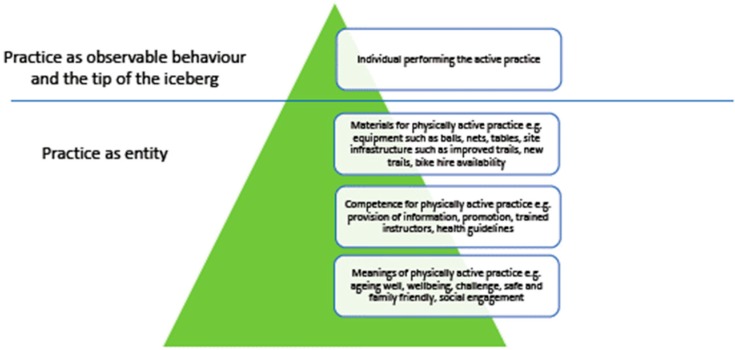
Adapted from Spurling et al. [26] and highlighting that physically activity behaviours are the tip of the iceberg.

This study contributes to the existing evidence base by taking a social practices perspective and qualitative approach; it highlights the importance of focusing beyond the individual in interventions that try to encourage physical activity behaviours. A lot of the existing research on behaviour change focuses on the individual and the choices and decisions they make to sustain or become more physically active or not [42,43,44,45]. A social practices perspective argues that individual behaviours are performances of social practices, with these individual behaviours being those actions that are observable. However, they are the expressions of wider social phenomenon such as meanings, materials and competence (Figure 5). Policies and interventions should target these areas otherwise they are potentially only focusing on the tip of the iceberg. By improving infrastructure for physical activity, providing opportunities to join regular physical activity sessions, promoting the programme in different ways to a variety of participants as well as providing suitable information and linking with trained instructors the Active Forests programme is changing the socially shared meanings of many physical activity practices that can be undertaken in a forest environment.

### 4.3. Forests as Spaces for Women to Be Comfortable in Being Physically Active

Some of the Active Forests programme activities are targeted specifically at women such as the Buggy Fit classes and Real Spin mountain biking for women. The Bootcamp classes while not specifically aimed at women were held at a time suitable for those dropping off their children at school in the morning. However, other activities seemed to appeal more to women such as the Nordic walking, which attracted more older women than men. Parkrun had a good mix of families and many women participants. The forest environment was seen by some of the women as a non-judgemental space, where they often felt less self-conscious and this was also reflected in the ethos of the various activities such as parkrun, Pilates and Nordic walking which were encouraging all types of people, of all abilities and shapes and sizes. This is critical, the Sport England [11] insight ‘go where women are’ highlights the importance of creating physical activity offers that suit women, using the term physical activity rather than sport which can have negative associations [46], and offering support sometimes through women only sessions, and allowing space for social interaction.

## 5. Conclusions

Although the overall research for the Active Forests programme involves mixed methods, this paper focuses on the qualitative data and provides insights into how people engage with the programme and how Forestry England and Sport England have recognised the need to focus on a number of elements to engage and encourage people to be active in a forest environment. Public forest managers could usefully take a social practices perspective when designing interventions that aim to engage and connect people with the forest environment. There is potential that this type of approach might also be more sustainable in terms of encouraging physical activity in the long term in the forest environment; as it provides infrastructure and equipment, links to wider socially shared meanings on healthy and active lifestyles, and provides instructors and volunteers that can help support participants to start being active, increase activity or remain active.

## Figures and Tables

**Table 1:** Key elements of the Active Forests Programme (AFP).

Key Elements of the AFP and Types of Physical Activities Offered	Examples
Changes and improvements to the existing forest site infrastructure	New or improved running, orienteering, cycling trails
Provision of equipment for activity	Table tennis tables and bats, goals and footballs, volleyball nets and balls, rounders bats and balls provided at some sites. This can also include the use of phone apps, e.g., a Gruffalo spotter app was developed so the users could follow clues such as a Gruffalo footprint, and when they find it a short animation of the character is triggered which blends in with the natural surroundings.
Organised and led regular weekly activities these: Are led by trained instructors or volunteers Take place on a regular basis, e.g., weekly—although they may stop during school holidays	Nordic walking, park run ^1^, Pilates, fitness, tai chi, archery, buggy fit, Bootcamp. There can be overlap between activities, e.g., running can be self-led or part of an event or part of a class led by an instructor.
Organised events (one off, sporadic or yearly events)	10 Km runs, duathlons, fun runs, cycle events, canicross ^2^, orienteering
Self-led activities	Table tennis, Gruffalo ^3^ orienteering, running, cycling, mountain biking, volleyball, rounders, badminton, cricket, football, tennis, walking
Communication and marketing	To publicise the programme and develop new opportunities to promote physical activity.

^1^ Parkrun organise a weekly Saturday 5 Km run at a set time (9 a.m.) across a range of sites in the UK and internationally. ^2^ Canicross is the sport of cross country running with dogs. ^3^ The Gruffalo is a monster character in a children’s book. Forestry England worked with the author and developed a trail for children to go and find a wooden sculpture of the Gruffalo located in the forest.

**Table 2:** Interviewee and focus group information from nine different forest sites.

Forest Location	Activity	Area of Country	Interview/Focus Group	Age Range of Participants	Male	Female	Employment Status	Disability or Limited in Daily Activities	Month/Year of Data Collection
Delamere Forest	Nordic walking	North west	Focus group	55–74	1	10	Working 1, Retired 10	None	October 2015
Sherwood Pines Forest	Parkrun	Midlands	Interviews and mini focus group	25–64	3	10	Retired 1 Employed 7 Self-employed 3 Student 1	1 person	February 2016
Bedgebury Forest	Real Spin (mountain biking for women)	South east	Focus group	35–54	0	9	Retired 1 Employed 3 Self-employed 1 Looking after family 4	None	February 2016
Cannock Chase Forest	Orienteering	Midlands	Focus group	13–75+	10	4	Not given	None	August 2016
Dalby Forest	Go Tri (duathlon)	North east	Interviews	25–64	9	5	No data	No data	February 2017
Haldon Forest	Nordic fitness walking	South west	Focus group	45–75+	1	10	Retired 8 Employed 2 Looking after family 1	7 persons	April 2018
Thetford Forest	Table Tennis	South east	Interviews	35–74	5	9	No data	No data	April 2018
Delamere Forest	Pilates	North west	Focus group	25–75+	3	5	Retired 5 Employed 1 Self-employed 2	None	April 2018
Whinlatter Forest	Buggy Fit	North west	Focus group	16–44	0	5	Employed 2 Looking after family 1 Maternity leave 2	None	May 2018
Dalby Forest	Canicross	North east	Interviews	16–64	3	8	No data	No data	September 2018
Alice Holt Forest	Bootcamp	South east	Focus group	35–54	0	10	Employed 2 Looking after family 4 Self-employed 4	None	June 2019
				Total	35 men	85 women			

**Table 3:** Benefits identified by participants.

Benefits	Responses from Participants and Observational Field Notes	Activity and Forest Site
Social	‘Because there are groups like the ‘run fit mums’ and this [Bootcamp] which is all so social as well’ ‘It all adds to being more supportive and encouraging for others to come along ‘It’s very social’. ‘Yes, you can have a chat’ ‘You have like-minded people, so you talk a lot’ ‘And then they are having an enjoyable day not just with their family but with strangers as well’ ‘Yes, we always go for a drink afterwards in the café, we try and make it a social and family activity as well’	Alice Holt Forest, Bootcamp Haldon Forest and Delamere Forest, Nordic walking Dalby Forest, Go Tri Sherwood Forest, Parkrun
Escape and freedom	Interviewer: what is it about being outside? ‘Freedom’ ‘We enjoy the feeling of freedom’ ‘I feel like a child again—freedom, it’s lovely’	Whinlatter Forest, Buggy Fit Bedgebury Forest, Real Spin
Learning and skills	‘It’s learning the different aspects of Nordic walking, how to use the poles, how to exercise, how to warm up and cool down and a bit of health and safety’	Delamere Forest, Nordic walking
Sense of achievement	‘It challenges you, it’s not something you do for a rest Nordic walking’	Delamere Forest, Nordic walking
Mental health	‘You can go at your own pace you’re not pressured into doing anything’	Delamere Forest, Nordic walking
Fresh air	‘The fresh air, the trees it is so good for us’ ‘But if you go for a walk you want to be breathing in fresh air, away from the traffic and diesel fumes’	Alice Holt Forest, Bootcamp Delamere Forest, Nordic walking
Physical	‘Trying to be healthier, lose weight in my case’ ‘I need to get back into it as well as I have had a health issue as well. I have just been diagnosed with rheumatoid arthritis, so he has cancer and I have this, so we are a right pair’	Sherwood Forest, Parkrun Dalby Forest, Go Tri
Forest environment	‘You’ve got the challenge of uneven terrain and some hills and then level ground That’s when you realise how good two poles are because it makes it so much easier walking with poles’ ‘We love the forest, know it well, have used it for many years. We have seen changes that have taken place in the forest over the past years. Now it is appealing to more diverse groups of people. We think the forest is beautiful, we are always seeing different things’	Delamere Forest, Nordic walking Dalby Forest, Go Tri

## Appendix A

The Eighteen Active Forests Sites, Location, Approximate Size and Some of the Key Activities Introduced through the Active Forests Programme.

**Table A1:** Active Forests programme site information.

**Site Name**	**Bedgebury Forest**	**Thetford Forest**	**Sherwood Pines Forest**	**Delamere Forest**	**Dalby Forest**
**Active Forests coordinator % of time spent on AFP** ^1^	80%	80%	80%	80%	80%
**Forest size in hectares approximately** ^2^	890	22,500	1500	1000	3575
**Forest type** ^3^	Includes the national pinetum and wider mixed species forest	Largest lowland pine forest in Britain including heaths and broadleaves	Pine forest	Mixed conifer and deciduous	Mixed conifer and deciduous
**Location**	Kent	On the Norfolk/Suffolk border	Nottinghamshire	Cheshire	North Yorkshire
**Some of the new organised events/activities developed**	Parkrun Archery, Table tennis Orienteering	Family fitness Gruffalo orienteering Cycling Table tennis Parkrun	Parkrun Gruffalo orienteering Football Volleyball Pilates Running	Bootcamp Running Family fitness Table tennis Orienteering Cycling	Netball, Nordic walking, Family fitness, Table tennis, Gruffalo orienteering
**Site Name**	**Birches Valley Cannock Chase**	**Forest of Dean**	**Salcey Forest**	**Alice Holt Forest**	**Haldon Forest Park**
**Active Forests coordinator**	80%	80%	80%	80%	80%
**Forest size in hectares approximately**	1200	7500	500	848	1725
**Forest type**	Mixed woodland	Mixed woodland Includes several different woods	Mixed woodland including ancient oaks	Mixed woodland	Mixed woodland. Includes several different woods
**Location**	Staffordshire	Gloucestershire	Northamptonshire	Surrey	Devon
**Some of the new organised events/activities developed**	Yoga, Archery, Volleyball, Table tennis, Mountain biking, Junior Parkrun	Nordic walking, Mountain biking, Football, Orienteering, Cycle events	Walking groups, Buggy Fit, Archery, Running events	Walking group Running group, Canicross, Buggy Fit, Bootcamp Orienteering	Yoga, Table tennis, Parkrun, Running events, Orienteering, Mountain biking, Fitness, Bootcamp

^1^ In the pilot phase of the programme Active Forests coordinators worked part time i.e., 50%. ^2^ Please note this is a very approximate estimate of the size of the forest sites, as they are not always discreet easy to measure areas. ^3^ Please note this is a very broad and approximate definition of forest type.

**Table A2:** Active Forests programme site information.

Site Name	Wendover Forest	Whinlatter Forest	Thames Chase	Chopwell Wood	Jeskyns Community Woodland
**Active Forests coordinator**	80%	80%	80%		
**Forest size in hectares approximately**	321	1200	41	375	150
**Forest type**	Mixed woodland	Mixed woodland	Community Woodland on former farm	Mixed woodland	New planted woodland on former farm.
**Location**	Buckinghamshire	Cumbria	London outskirts (urban)	Gateshead (urban)	Kent (urban)
**Some of the new organised events/activities developed**	Walking groups, Volleyball, Table tennis, Running events, Pilates, Buggy Fit, Parkrun	Walking group, Running events, Pilates, Orienteering, Climbing	Type II diabetes sessions and walking, Running events	Holiday hunger activities,	Running events, Children’s glow trail

**Table A3:** Active Forests programme site information.

Site Name	Hamsterley	Wyre Forest	Westonbirt
**Active Forests coordinator**	80%	80%	80%
**Forest size in hectares approximately**	2200	1915	242
**Forest type**	Mixed woodland	Mixed woodland	The National Arboretum. Picturesque parkland landscape
**Location**	County Durham (peri-urban)	Worcestershire	Gloucestershire
**Some of the new organised events/activities developed**	Running events, Children’s glow trail	Walking group, Volleyball, Table tennis, Running group, Nordic Walking, Buggy Fit, Canicross	Yoga, Tai Chi, Table tennis, Nordic Walking, Duathlon

## Appendix B. Active Forests Topic Guide for Focus Groups/Interviews

**Topic Guide for Focus Groups/Interviews with Users and User Groups**

**Introductions and Purpose of the Research**

Outline who the researcher is, the purpose of the research, the researcher commissioner, the voluntary participation of respondents, maintaining anonymity etc.

**Getting Involved in Physical Activity**

- When did you first got involved in sport and physical activity [prompt: what age. What activities]
- What motivated you to get involved in sport and physical activity [prompt: fitness, health, enjoyment,]
- How you got it involved [on your own, at school, joining an organised activity, going to an event, joining a club]
- How regularly do you undertake physical activity [everyday, every week, month]
- Do you consider yourself to be a sport and exercise type of person [prompt: do you view [insert name of sport] as a sport?
- Do you think you are meeting the recommendation of 150 min of a mix of moderate and vigorous activity per week? [could include active travel, gardening as well as leisure activity]

**Getting Involved in Physical Activity in Woods**

- When did you first get involved in physical activity in a woodland environment (Prompt: what type of activities do you do in woodlands)
- Has anyone not done this [name activity] type of activity before?
- What motivated you to do this?
- How regularly do you do this activity in a forest environment?
- Do you do this and/or other physical activity in other outdoor places (non-woodland i.e., parks, country parks, countryside, etc.)?
- What is the balance between activity you do in the forest/outdoors and in other places?
- What do you base your decisions on concerning whether you do your exercise in the forest or elsewhere?

**Experience of Exercise in the Forest**

- What is your experience of undertaking physical activity in a woodland?
- What are the benefits—are they different from doing sport in other locations—sports centres/gyms/sports fields? [prompt: fresh air, sensory stimulation, views, challenges—see next question]
- What are the challenges [prompt: weather, facilities, getting to woodland, uneven terrain, getting to location, cost of parking and events]
- Is there anything that would make it easier for you to do physical activity in a woodland [prompt: facilities, organised activities, infrastructure improvement i.e., better trails]

**Behaviours (Sustaining and Changes)**

- Has participating in this physical activity in this woodland led to any changes in your behaviour? [prompt: doing a new activity, sustaining behaviour in long term, changing behavior—doing more or different types of activity, doing more exercise overall, other such as encouraging a friend to join you].
- Has getting involved in this physical activity in this forest led you to visit it more often [prompt: how often—once a week more, once a month more etc.]
- How important is it for you to join an organised exercise activity in woodland that is led by someone [prompt: if it is important why is that the case, would you still do it if you were on your own]

**Social Connections**

- How important to you is undertaking physical activity in woodlands with others whether family, friends etc.
- Have you met new people through this activity, does this play any part in motivating you to attend or do you do activity with friends/family?
- What is your experience of the social side of this activity, would you do the activity alone?

**Experience of the Physical Environment**

- What is the importance or not of different aspects of woodlands—visual (what you see), sound (what you hear), smell (what you smell), texture (textures you see or feel by hand or underfoot) to your experience and any impact on your health and wellbeing.
- Is there anything specifically enables you to use this wood and others—[prompt: it’s nearby, organised activities, having dog to walk, going with someone else/company, familiarity with site, meeting new people, personal motivation]

Is there anything else you would like to say in relation to physical activity in woodlands

## References

[1] British Heart Foundation (2017). Physical Inactivity and Sedentary Behavior—Report 2017.

[2] Public Health England (2019). Physical Activity: Applying All Our Health.

[3] Heath G.W., Parra D.C., Sarmiento O.L., Andersen L.B., Owen N., Goenka S., Montes F., Brownson R.C. (2012). Evidence-based intervention in physical activity: Lessons from around the world. Lancet.

[4] National Institute for Health and Care Excellence (NICE) (2012). Physical Activity: Walking and Cycling—Public Health Guideline PH41.

[5] Gerber M., Puehse U. (2009). Review article: Do exercise and fitness protect against stress-induced health complaints? A review of the literature. Scand. J. Public Health.

[6] De Mello M.T., Vde Lemos A., Antunes H.K., Bittencourt L., Santos-Silva R., Tufik S. (2013). Relationship between physical activity and depression and anxiety symptoms: A population study. J. Affect. Disord..

[7] Hegberg N.J., Tone E.B. (2015). Physical activity and stress resilience: Considering those at-risk for developing mental health problems. Ment. Health Phys. Act..

[8] Molanorouzi K., Khoo S., Morris T. (2015). Motives for adult participation in physical activity: Type of activity, age and gender. BMC Public Health.

[9] Egli T., Bland H.W., Melton B.F., Czech D.R. (2011). Influence of Age, Sex, and race on college Students’ exercise motivation of physical activity. J. Am. Coll. Health.

[10] Hoare E., Stavreski B., Jennings G.L., Bronwyn A., Kingwell A. (2017). Exploring motivation and barriers to physical activity among active and inactive Australian adults. Sports.

[11] Sport England Go Where Women Are: Insight in Engaging Women and Girls in Sport and Exercise.

[12] Gavin J., Keough M., Abravenel M., Moudrakovskil T., McBrearty M. (2014). Motivations for participation in physical activity across the lifespan. Int. J. Wellbeing.

[13] Caperchione C., Vandelanotte C., Kolt G. (2012). What a man wants: Understanding the challenges and motivations to physical activity participation and health eating in middle-aged Australian men. Am. J. Men’s Health.

[14] WHO European Regional Office (2015). Infographic: Make Physical Activity a Part of Daily Life during All Stages of Life. http://www.euro.who.int/en/health-topics/disease-prevention/physical-activity/data-and-statistics/infographic-make-physical-activity-a-part-of-daily-life-during-all-stages-of-life.

[15] Thompson Coon J., Boddy K., Skein K., Whear R., Barton J., Depledge M. (2011). Does participating in physical activity in outdoor natural environments have a greater effect on physical and mental wellbeing than physical activity indoors? A systematic review. Environ. Sci. Technol..

[16] Public Health England (2014). Everybody Active, Everyday: An. Evidence Based Approach to Physical Activity.

[17] Hartig T., Mitchell R., de Vries S., Frumkin H. (2014). Nature and health. Annu. Rev. Public Health.

[18] Hunter R., Christian H., Veitch J., Astell-Burt T., Schipperijn J. (2015). The impact of interventions to promote physical activity in urban green space. A systematic review and recommendations for future research. Soc. Sci. Med..

[19] Jansen F.M., Ettema D.F., Kamphuis C.B.M., Pierik F.H., Dijst M.J. (2017). How do type and size of natural environments relate to physical activity behavior?. Health Place.

[20] Kondo M.C., Jacoby S.F., South E.C. (2018). Does spending time outdoors reduce stress? A review of real-time stress response to outdoor environments. Health Place.

[21] Mitchell R. (2013). Is physical activity in natural environments better for mental health than physical activity in other environments?. Soc. Sci. Med..

[22] Schipperjin J., Bentsen P., Troelsen J., Toftager M., Stigsdotter U. (2013). Associations between physical activity and characteristic of urban green space. Urban For. Urban Green..

[23] O’Brien L., Morris J. (2014). Well-being for all? The social distribution of benefits gained from woodlands and forests in Britain. Local Environ..

[24] Sport England (2015). Getting Active Outdoors: A Study of Demography, Motivation, Participation and Provision in Outdoor Sport and Recreation in England.

[25] O’Brien L., Forster J. (2017). Fun and Fitness in the Forest: Monitoring and Evaluation of the Three Year Active Forests Pilot Programme.

[26] Spurling N., McMeekin A., Shove E., Southerton D., Welch D. Interventions in Practice: Re-Framing Policy Approaches to Consumer Behaviour; Sustainable Practices Research Group: 2013. http://www.sprg.ac.uk/uploads/sprg-report-sept-2013.pdf.

[27] Shove E. (2010). Beyond the ABC: Climate change policy and theories of social change. Environ. Plan. A Econ. Space.

[28] Shove E., Pantzar M., Watson M. (2012). The Dynamics of Social Practice: Everyday Life and How It Changes.

[29] O’Brien L., Morris J., Stewart A. (2014). Engaging with peri-urban woodlands in England: The contribution to people’s health and well-being and implications for future management. Int. J. Environ. Res. Public Health.

[30] Morris J., O’Brien L. (2011). Encouraging healthy activity amongst under-represented groups: An evaluation of the Active England woodland projects. Urban For. Urban Green..

[31] Maas J., van Dillen S., Verheij R., Groenewegen P. (2009). Social contacts as a possible mechanism behind the relation between green space and health. Health Place.

[32] O’Brien L., Forster J. (2020). Sustaining and changing physical activity behaviours in the forest: An evaluated pilot intervention on five public forest sites in England. Urban For. Urban Green..

[33] Archibald D., Douglas F., Hoddinott P., Van Teijlingen E., Stewart F., Robertson C., Boyers D., Avenell A. (2015). A qualitative evidence synthesis on the management of male obesity. BMJ Open.

[34] Lewis K., Fraser C., Manby M. (2014). ‘Is it worth it?’ A qualitative study of the beliefs of overweight and obese physically active children. J. Phys. Act. Health.

[35] Social and Economic Research Group (2010). SERG Research Ethics.

[36] Braun V., Clarke C. (2006). Using thematic analysis in psychology. Qual. Res. Psychol..

[37] Coleman L., Cox L., Roker D. (2007). Girls and young women’s participation in physical activity: Psychological and social influences. Health Educ. Res..

[38] Marcu A., Uzzell D., Barnett J. (2011). Making sense of unfamiliar risks in the countryside: The case of Lyme disease. Health Place.

[39] O’Brien L., Marcu A., Marzano M., Barnett J., Quine C.P., Uzzell D. (2012). Situating risk in the context of a woodland visit: A Case Study on *Lyme Borreliosis*. Scott. For..

[40] Bosteder S.M., Appleby K.M. (2015). Naturally fit: An investigation of experiences in a women only outdoor recreation program. Women Sport Phys. Act..

[41] Twohig-Bennett C., Jones A. (2018). The health benefits of the great outdoors: A systematic review and meta analysis of greenspace exposure and health outcomes. Environ. Res..

[42] Ajzen I., Kuhl J., Beckman J. (1985). From intentions to actions: A theory of planned behaviour. Action-Control: From Cognition to Behaviour.

[43] Ajzen I. (1991). The theory of planned behavior. Organ. Behav. Hum. Decis..

[44] Hardeman W., Johnston M., Johnston D.W., Bonetti D., Wareham N.J., Kinmonth A.L. (2002). Application of the Theory of Planned Behaviour in Behaviour Change Interventions: A systematic review. Psychol Health.

[45] Dolan P., Hallsworth M., Halpern D., King D., Metcalfe R., Vlaev I. (2010). MINDSPACE: Influencing Behaviour through Public Policy.

[46] Cardinal B., Yan Z., Cardinal M. (2013). Negative experiences in physical education and sport: How much do they affect physical activity participation later in life?. J. Phys. Educ. Recreat. Dance.

